# Beyond sensory conflict: The role of beliefs and perception in motion sickness

**DOI:** 10.1371/journal.pone.0245295

**Published:** 2021-01-19

**Authors:** Suzanne A. E. Nooij, Christopher J. Bockisch, Heinrich H. Bülthoff, Dominik Straumann

**Affiliations:** 1 Department of Human Perception Action and Cognition, Max Planck Institute for Biological Cybernetics, Tübingen, Germany; 2 TNO Soesterberg, Soesterberg, The Netherlands; 3 Department of Neurology, University Hospital Zurich & University of Zurich, Zurich, Switzerland; 4 Department of Ophthalmology, University Hospital Zurich & University of Zurich, Zurich, Switzerland; 5 Department of Otorhinolaryngology, University Hospital & University of Zurich, Zurich, Switzerland; 6 Interdisciplinary Center for Vertigo & Neurological Visual Disorders, University Hospital Zurich & University of Zurich, Zurich, Switzerland; 7 Clinical Neuroscience Center, University Hospital Zurich, Zurich, Switzerland; 8 Swiss Concussion Center, Zurich, Switzerland; Tokai University, JAPAN

## Abstract

Illusory self-motion often provokes motion sickness, which is commonly explained in terms of an inter-sensory conflict that is not in accordance with previous experience. Here we address the influence of cognition in motion sickness and show that such a conflict is *not* provocative when the observer believes that the motion illusion is indeed actually occurring. Illusory self-motion and motion sickness were elicited in healthy human participants who were seated on a stationary rotary chair inside a rotating optokinetic drum. Participants knew that both chair and drum could rotate but were unaware of the actual motion stimulus. Results showed that motion sickness was correlated with the discrepancy between participants’ *perceived* self-motion and participants’ *beliefs* about the actual motion. Together with the general motion sickness susceptibility, this discrepancy accounted for 51% of the variance in motion sickness intensity. This finding sheds a new light on the causes of visually induced motion sickness and suggests that it is not governed by an inter-sensory conflict per se, but by beliefs concerning the actual self-motion. This cognitive influence provides a promising tool for the development of new countermeasures.

## Introduction

There is a growing body of evidence showing the bi-directional interactions between cognition and vestibular processing (see [1] for a review). For example, self-motion perception, and even ocular motor reflexes are affected by mental imaginary [2, 3], or by prior expectations of the motion stimulus [4, 5]. The cognitive context of the motion stimuli is also known to affect motion perception, as has been shown for the compelling sense of self-motion that can be induced by moving visual, or to a lesser extent, auditory stimuli (i.e., vection). This self-motion illusion is stronger and/or occurs faster if the moving stimuli are associated objects that are normally stable in the environment and the participant is positioned on a device that allows actual motion [6–8]. Here we explore the effects of cognition on a different aspect of vestibular processing, namely motion sickness. If motion sickness can be influenced by the cognitive aspects described above, this would offer new methods to counteract this malady.

Motion sickness is the unwanted side-effect of passive transportation, with pallor, cold sweating, nausea and vomiting being its cardinal signs [9]. It negatively affects training in virtual environments or simulators and is recognized as a potential problem in automated vehicles [10]. Although other theories exist (see [11, 12] for reviews), situations provoking motion sickness are most often explained in terms of “inter-sensory conflicts”, i.e. when information from the different sensory systems is combined in an unusual way [13]. In the case of vection for example,—which is often used as a stimulus to evoke motion sickness [14–16]—the motion information provided by vision is not corroborated by the vestibular system, and this incongruency is often considered as the cause of motion sickness. This inter-sensory conflict theory was refined by Reason and Brand [9, 17] who argued that such a conflict alone is not sufficient to provoke motion sickness, but that the decisive mechanism includes a comparison between the current, unfamiliar sensory pattern and what is expected based on previous experience. This is termed the sensory re-arrangement theory and its corollary, the neural mismatch model [17]. By including the comparison with previous experience (also called *exposure history* or *neural store*), the theory is able to account for the fact that motion sickness symptoms generally disappear with repeated exposure, that is, when the unfamiliar sensory pattern is becoming the norm. As a result, a discrepancy among sensory inputs only provokes motion sickness if the sensory pattern is not part of expectations based on the recent exposure history.

Expectations thus form a key element in motion sickness. Here, we explored whether the “expected sensory feedback” is affected by factors other than the exposure history. Motion illusions, such as vection, are, by definition, situations of inter-sensory conflict, and prolonged exposure indeed often leads to motion sickness [14–16]. By the same token, however, motion illusions are characterized by a discrepancy between what is perceived and what is actually happening. We propose that this discrepancy plays a key role in motion sickness evoked by motion illusions. More specifically, we hypothesize that motion sickness induced by visual motion in stationary observers is not due to the unfamiliarity to the incongruent stimulus pattern, but to a discrepancy between what is perceived and what is believed to be actually happening. Knowledge on–or beliefs about–the actual situation represents a cognitive influence that may shape the expected sensory feedback, and thereby affect motion sickness.

This hypothesis was based on previous work, where we investigated factors contributing to motion sickness induced by visual yaw rotation [18]. Here, participants were seated in the center of a large 180 deg wide projection dome, where the projected scene rotated around them about an earth-vertical axis. This stimulus induced reflexive eye movements (optokinetic nystagmus), and a compelling sense of vection. Prolonged, i.e. 20 min., exposure induced motion sickness. The study was designed to investigate the contribution of vection and optokinetic nystagmus to motion sickness, and to rule out the possibility that sickness occurred as the consequence of small inadvertent head movements that may have induced pseudo-coriolis effects [19]. As the vestibular system is sensitive to accelerative stimuli and not to constant velocity rotation, the inter-sensory conflict theory would predict that motion sickness was affected by the *changes* in vection (e.g. perceived rotation speed), when a vestibular signal is expected. The results, however, showed that not changes in vection, but the overall vection level itself correlated with motion sickness intensity: visual conditions that induced stronger vection also induced more motion sickness. As a possible explanation for these results, we hypothesized that the sickness was not provoked by the motion illusion itself, but by the fact that participants were aware of the fact that the perceived self-motion was not actually occurring, as the setup was clearly not capable of it. From this, it follows that a motion illusion is only sickening if it contradicts with beliefs about the true motion, but not when beliefs and perception coincide. If this cognitive influence is indeed responsible for the observed motion sickness, cognitive mechanisms could be utilized to manipulate motion sickness.

To investigate the possible involvement of beliefs about the actual motion in visually induced motion sickness, the previous study was repeated in a setup that is likely to elicit a belief of actual self-motion, namely an optokinetic drum surrounding a rotary chair. Both drum and chair are motorized, rotate about the same earth-vertical axis and can move independently from one another. This setup causes that, when rotating at constant velocity, drum rotation about the stationary chair is indistinguishable from chair rotation inside the stationary drum. Thus, in such a setup any perceived, visually induced self-motion could be attributed to physical self-motion, and not to surround motion. While leaving participants unaware of the actual motion stimulus, we investigated whether variation in motion sickness levels between participants could be explained by variation in perception of and beliefs about self-motion, and the discrepancy between the two.

## Methods

### Participants

**Fig 1:**
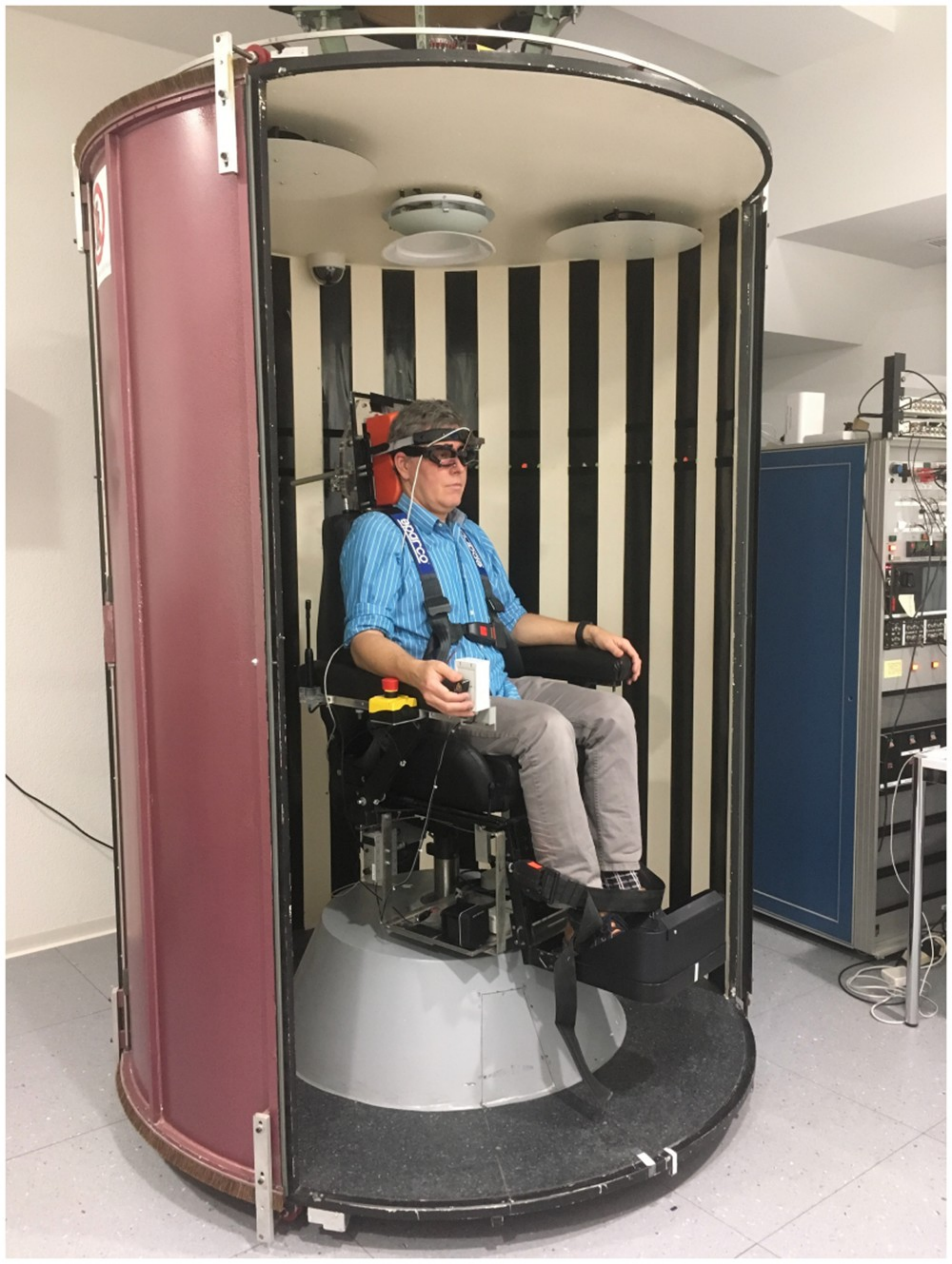
Experimental set up with motorized chair and drum that could rotate independently from each other. During the test the surrounding drum was closed and rotating around the stationary chair.

Twenty healthy volunteers (12 males, 8 females) with a mean age of 22 years (range 20–25) were invited to participate in the study. All were free from any neurological or vestibular disorder and had normal or corrected to normal vision with the aid of contact lenses (wearing glasses was not possible in the experimental setup). People suffering from claustrophobia were discouraged from participating, as this might affect the motion sickness response [20]. Participants were excluded if they had no previous experience with motion sickness, as assessed by the Motion Sickness Susceptibility Questionnaire—Short (MSSQ [21]). This occurred in one volunteer, leaving 19 participants in total. The average MSSQ score of this group was 16.6 (range 2.0–48.0), which is equivalent to the 55^th^ percentile of a reference population [22]. All participants gave written informed consent prior to any data collection. The person depicted in Fig 1 has given written informed consent (as outlined in the PLOS consent form) to publish the picture. The study was approved by a local ethics committee (Kantonale Ethik-Kommission Zurich) and performed according to the ethical guidelines laid out in the Declaration of Helsinki.

### Experimental set-up

The study was performed in the optokinetic drum of the University Hospital Zurich, Switzerland. This experimental device (Tönnies, Jaeger, Germany) consists of a motorized chair inside an enclosed cylindrical drum (diameter = 1.44 m, height 2.03 m), where both chair and drum can be rotated independently about the central, earth-vertical axis (Fig 1). The inside of the drum is covered with alternating vertical black and white stripes (width 0.1 m). The chair is equipped with safety belts around the torso and lower legs, and with arm and leg rests to ensure a comfortable, upright body position. The participant’s head was supported by a Sinmed contoured foam headrest (CIVCO Medical Solutions, Kalona IA, USA) with additional Velcro straps around the forehead to keep the head upright and stationary. A rotatory knob attached to the right arm-rest was used by the participant to indicate perceived self-motion. Eye and head movements were measured at a frequency of 220 Hz using a head mounted, video-based eye tracking system with an integrated inertial measurement unit (EyeSeeCam^®^, Autronic Medizintechnik, Germany).

### Procedures

Prior to the test, participants were told that they were participating in a study investigating the relationship between motion perception and motion sickness. They were shown that drum and chair could rotate independently, but they were kept unaware of the actual motion stimulus to which they would be exposed. In this way, we choose not to explicitly manipulate the beliefs about the actual motion stimulus, but to let it freely emerge from the experimental setup. After giving written informed consent, they completed the MSSQ [21] to assess their general susceptibility to motion sickness.

During the test, the participant was positioned in the chair and equipped with the eye tracker goggles. With lights on, the drum was accelerated with 10 deg/s^2^ to a constant speed of 60 deg/s. The participant was asked to look approximately straight ahead, without actively following the individual stripes. Furthermore, the participant was asked to report whether self-motion was perceived using the knob on the right armrest. The knob could be rotated in the sagittal plane over a range of 90 deg and indicated the ratio between perceived self- and drum-motion [18, 23]. One endpoint meant “I perceive myself as stationary and the drum as rotating” and the other “I perceive myself as rotating and the drum as stationary”. Turning the knob away from an endpoint indicated that the ratio between perceived self- and drum motion changed, and that both were perceived as rotating. In the remainder of the manuscript, the endpoints will be referred to as “no vection” and “full vection”, respectively. To monitor motion sickness during the test, its intensity was verbally reported by the participant every two minutes (indicated by a beep) using the Fast Motion Sickness scale (FMS [24]). This numerical scale focusses on the nausea aspect of motion sickness, where 0 means “no symptoms” and 20 indicates “frank sickness”. The motion stimulus was terminated after 20 min, or when an FMS score of 15 was reached. Once the motion had stopped, participants answered the question “Are you motion sick (Y/N)?” and completed the Simulator Sickness Questionnaire (SSQ [25]). The latter contains a list of 16 motion sickness symptoms that were rated on a 4-point scale. From these ratings, a Total Score (TS) for motion sickness intensity was calculated. Then the participant was asked about his/her beliefs regarding the actual motion stimulus using the following questions: “Did the chair actually rotate the whole time (Y/N) and how sure are you of your answer?” and “Did the drum actually rotate the whole time (Y/N) and how sure are you of your answer?”. Certainty scores were obtained using a visual analogue scale ranging from “very uncertain” to “very certain”.

### Data analysis

The data on perceived self-motion was expressed as a fraction between 0 and 1, where all data points below 0.05 were categorized as “no vection” and all data points larger than 0.95 as “full vection”. The strength of perceived self-motion was characterized by the percentage of the time spent in full vection (FullVection). Changes in the vection rating over time were characterized by calculating the standard deviation of the first derivative of the self-motion rating (SDdV) [18]. Regarding the beliefs about actual motion stimulus, the binary Y/N answer (“Did the chair/drum actually rotate the whole time?”) and the certainty score were combined into a single Rotation Likelihood (RL) for drum and chair separately. To that end, certainty scores (ranging from 0 to 1 on the visual analogue scale) were first signed negative for ‘N’ and positive for ‘Y’, resulting in intermediate scores ranging from -1 to +1. These were subsequently remapped on a range between 0 (“I am very certain that no continuous rotation occurred”) and 1 (“I am very certain that continuous rotation occurred”), where 0.5 indicated maximal uncertainty about the actual motion stimulus. The difference between the perceived and believed motion (Conflict_PB_) was operationalized as the absolute difference between the fraction of the time spent in full vection (FullVection/100) and the rotation likelihood of the chair (RL_chair_). Both these variables have a range from 0 to 1, so the resulting conflict score also ranged between 0 (perception and belief identical) and 1 (perception and belief divergent).

Optokinetic nystagmus and inadvertent micro head movements were also recorded during the test as control measures, as these have been recognized as potential contributors to motion sickness induced by visual rotation [18, 19, 26]. To obtain an estimate of the slow-phase eye velocity (SPEV) of the optokinetic nystagmus, horizontal eye velocity and acceleration were calculated from the eye position data, using a Gaussian low-pass filter and a numerical three-point differentiation. Fast phases were detected using the algorithm of Behrens & Weiss [27] with an acceleration threshold of 1200 deg/s^2^, and removed from the eye velocity signal. Subsequently, the desaccaded eye velocity signal was filtered using a 0.25 s window median filter to obtain the SPEV over time. The median SPEV was taken to represent the average eye behavior. To verify whether any inadvertent micro head movements occurred during the test–which could have caused nauseating pseudo-coriolis effects [19]–the head angular velocity data was low pass filtered (cut-off frequency of 10 Hz), and we identified the number of instances where the Earth-horizontal component of the head angular velocity exceeded a value of 3 deg/s (nHM [18]). All data processing was performed with Matlab R2015b (The Mathworks Inc., United States).

Two-tailed Spearman correlation coefficients between motion sickness intensity, as characterized by the SSQ Total Score (SSQ-TS), and the various predictor variables were calculated to asses which variables were most suitable as predictors in a multiple regression model. In this regression analysis the dependent variable SSQ-TS was transformed using a logit function to ensure homogeneity of variances in the residuals. Influential cases were identified by analyzing the externally studentized residuals and computing the so-called DFFITS index [28]. Observations for which the absolute value of the externally studentized residual exceeded 1.96 or with an absolute DFFITS value exceeding 1 were excluded [29]. The statistical analyses were performed in R, version 3.5.1 [30].

## Results

To examine whether motion sickness is the result of a discrepancy between perception of self-motion and beliefs, we first needed to test whether the preconditions for the testing of such an hypotheses were met: Did participants indeed experience self-motion? Did they vary in the belief about what was actually happening? And did participants indeed turn sick? In the following we will first discuss these variables separately and subsequently examine relationships between them.

All participants experienced a state of full vection during at least a portion of the trial. In one participant this percept remained stable over the course of the trial, all others switched multiple times between states of no vection and states of full vection. Intermittent levels were of relatively short duration. This switching pattern was qualitatively similar to the vection patterns observed in our previous study [18]. The average percentage of the time spent in full vection was 44% (SD = 21) and the time spent in no vection 30% (SD = 18). In the following, the percentage of time spent in full vection (FullVection) was taken as the measure for vection intensity.

**Fig 2:**
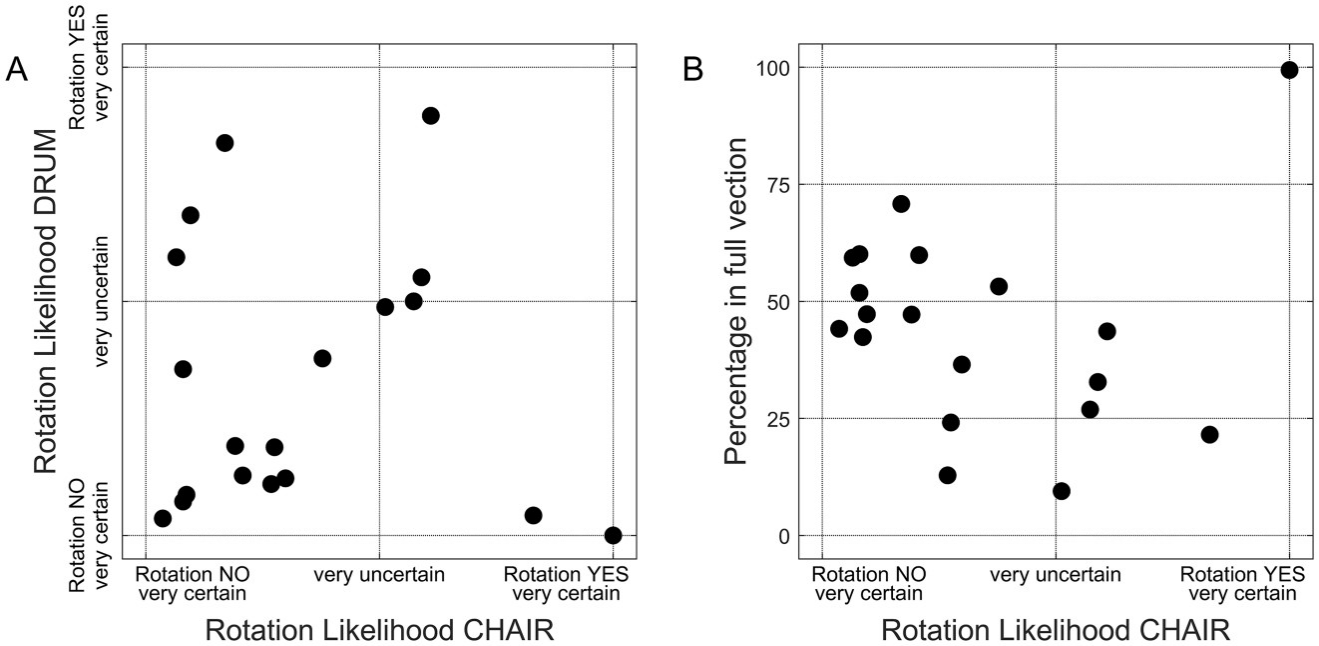
Scatterplots of A) chair rotation likelihood against drum rotation likelihood (n = 19); B) chair rotation likelihood against and Fullvection (i.e., percentage of time spent in full vection, n = 19).

Fig 2A depicts the individual data on the beliefs about the actual motion: the rotation likelihood scores of chair (RL_chair_) and drum (RL_drum_). For the chair, 6 out of 19 participants answered affirmative to the question whether the chair was rotating the whole time, with different levels of certainty. For the drum, this was the case in 5 participants. These responses partly contradict the vection data on self-motion perception, showing that all but one of the participants perceived periods of both drum stationarity and chair stationarity. Thus, this contradiction suggests that the participants realized that their beliefs about the actual motion stimulus could diverge from how they had perceived the motion.

Fig 2B shows the relationship between the beliefs about actual self-motion and the percentage of time spent in full vection. The participant who experienced full vection during the whole stimulus was indeed very certain that the chair rotated the whole time. For the others, the relationship between vection intensity and beliefs was negative: (*ρ* = -0.58, p = 0.0094, n = 18): an increase of the chair rotation likelihood was associated with lower values for FullVection. This appears to be different from what is found in other studies [6, 8], and suggests that participants were aware of the fact that the experimental setup allowed for a discrepancy between the actual motion and how it was perceived. We will elaborate on this in the Discussion section.

We found that the exposure to the optokinetic stimulus caused symptoms of motion sickness in the majority of participants, with 11 out of 19 regarding themselves to be motion sick. The SSQ-TS scores ranged between 37 and 161 (on a 236-point scale), where the median score was 58 (Median Absolute Difference, MAD = 6) for the non-sick group, 105 (MAD = 26) for the sick group and 79 (MAD = 22) overall. This variation in responses was also reflected in the FMS scores obtained during the exposure. FMS scores ranged between 1 and 15, where five participants stayed below FMS 5, six had intermediate scores between 5 and 9, and eight participants ended with scores of 10 or higher. Three participants reached the stop criterium FMS 15 and aborted the trial prematurely. One of them, however, reported afterwards that no motion sickness had occurred, and also scored very low on the SSQ. As discrepancies between the FMS and SSQ scores were observed in two other participants, the overall correlation between FMS and SSQ-TS score was only 0.44 (p = 0.06). This is much lower than what is found in other studies [18, 24] and suggests that not all participants understood the usage of the scale. Therefore, the SSQ-TS score was used as a measure for motion sickness intensity in the remaining analysis.

### Relation between motion sickness and the discrepancy between perception and beliefs

**Fig 3:**
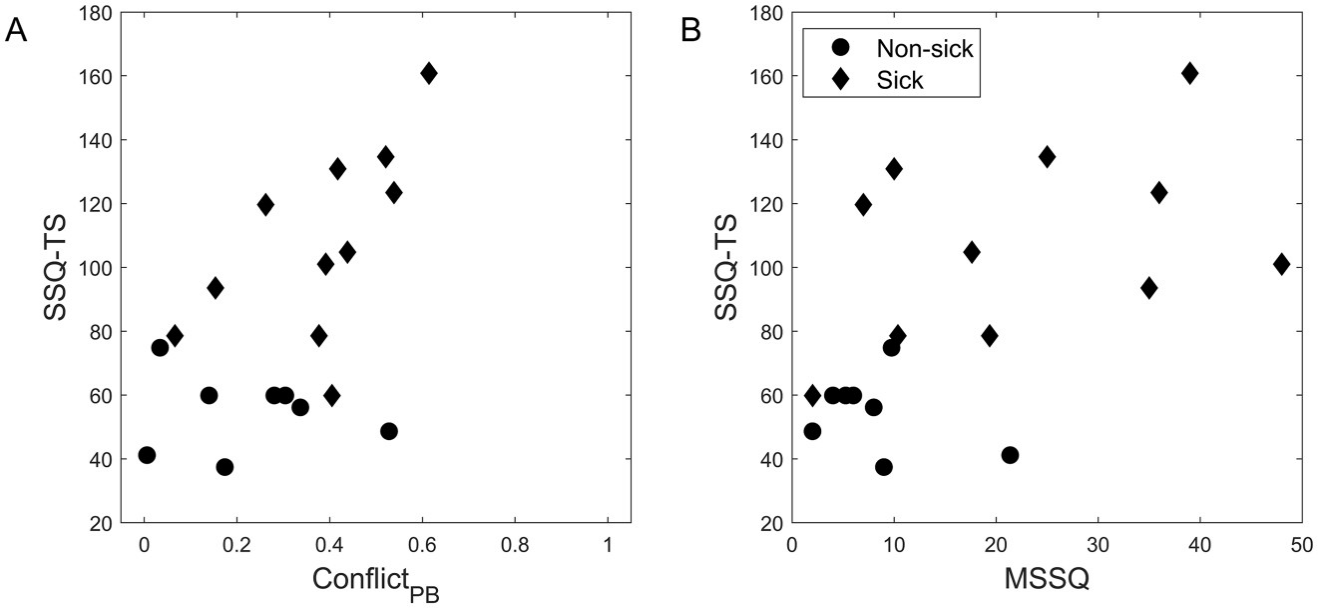
Scatterplots of conflict_PB_ (A) and MSSQ (B) vs. motion sickness intensity (SSQ-TS, n = 19). Diamonds and circles indicate the sick and non-sick group resp., according to self-judgement.

According to our hypothesis, motion sickness should be correlated with the difference between perception and beliefs about actual motion. A conflict measure, Conflict_PB_, was defined as the absolute difference between the fraction of time in full vection (FullVection/100), and the chair rotation likelihood (RL_chair_). In a first exploratory analysis, we calculated spearman correlation coefficients between motion sickness intensity (SSQ-TS) and possible predictor variables. Next to Conflict_PB_, the fraction of the time spent in full vection (FullVection) and the rotation likelihood of chair (RL_chair_), these included the rotation likelihood of the drum (RL_drum_), the certainty of chair and drum rotation, the overall motion sickness susceptibility (MSSQ), the variability in the vection rating (SDdV), the average slow phase eye velocity (SPEV) and the number of inadvertent micro head movements (nHM). Although none of the correlations reached the level of significance after the Bonferroni correction for multiple comparisons, two of them clearly stood out and showed the largest correlations (Table 1). These were Conflict_PB_ (Fig 3A) and the overall motion sickness susceptibility (MSSQ, Fig 3B). Thus motion sickness intensity at the end of the trial increased with overall susceptibility to motion sickness, but also depended on how the motion stimulus was experienced. Not the experience of vection itself, or changes therein, was correlated with motion sickness, but the discrepancy between what was perceived and what was believed. In line with this interpretation, the one participant who contributed the perceived self-rotation entirely to actual chair motion did *not* develop sickness, despite having a higher than average motion sickness susceptibility.

**Table 1:** Spearman correlation coefficients and p-values for the relation between various predictor variables and SSQ-TS (n = 19). The last column shows p-values with Bonferroni correction for multiple comparisons.

	r	p	p_adjust_
**MSSQ**	0.60	0.007	0.066
**Conflict_PB_**	0.57	0.025	0.247
**RL_chair_**	0.01	0.960	1
**RL_drum_**	-0.01	0.971	1
**Certainty chair**	-0.19	0.446	1
**Certainty drum**	-0.17	0.477	1
**FullVection**	-0.07	0.761	1
**SDdV**	0.30	0.210	1
**SPEV**	0.07	0.774	1
**nHM**	-0.34	0.160	1

MSSQ = general motion sickness susceptibility; Conflict_PB_ = discrepancy between perceived and believed self-motion; RL_chair_ = Rotation Likelihood chair; RL_drum_ = Rotation Likelihood drum; Certainty chair = certainty score of chair rotation; Certainty drum = certainty score of drum rotation; FullVection = percentage of time spent in full vection; SDdV = measure for variability in vection rating; SPEV = median slow phase eye velocity; nHM = number of inadvertent head movements).

Subsequently, we assessed the portion of explained variance that could be attributed to these variables, using multiple regression. As stated in the Method section, influential cases were identified and excluded from the model, which occurred in 2 of the 19 cases and concerned cases with low MSSQ values. With the remaining 17 cases included, a model based on MSSQ only accounted for 37% of the variance in SSQ-TS, and adding Conflict_PB_ significantly improved the model fit (*F*(1,14) = 12.93, *p* = 0.003) and increased the explained variance to 67%. Fit parameters are summarized in Table 2. Adding more parameters to the model did not lead to further improvements. Applying this model on the total population (n = 19) resulted in an overall explained variance of 51%.

**Table 2:** Model parameters for the model describing motion sickness intensity (SSQ-TS, logit-transformed) by general motion sickness susceptibility (MSSQ) and the conflict between perceived and believed self-motion (Conflict_PB_).

	Estimate	SE	Standardized estimate	p
**(Intercept)**	-1.64	0.217	-	>0.001
**MSSQ**	0.02	0.007	0.42	0.019
**Conflict_PB_**	2.15	0.598	0.58	0.003

## Discussion

Earlier studies showed that motion perception is influenced by prior beliefs about the motion [4–6]. On the other hand, motion illusions, like vection, can be so powerful that they occur despite the fact the observer *knows* it is an illusion. The results of the current study suggest that this discrepancy between what is perceived and what is believed to be occurring, plays a leading role in the motion sickness that is often the byproduct of such motion illusions.

By using an optokinetic drum that allowed independent rotation of the participant and of the visual surround, participants knew that physical motion was possible, but were unaware of the actual motion stimulus. The rotation of the visual surround induced periods of perceived self-rotation in all participants, but the majority showed multiple switches between experiencing full, partial, or no vection. This is similar to the results of our previous study where the setup was not capable of physical rotation [18]. As the viewing behavior (e.g., voluntarily suppression of the optokinetic nystagmus) is known to affect vection [31], such perceptual switches could have been accompanied by changes in the slow phase eye velocity. Visual inspection of the recorded eye movement data, however, did not show any correspondence between the temporal evolvement of vection and eye velocity. Hence, we speculate that the switches in the perceptual state could have caused by the fact that participants were aware that both drum and chair could rotate. Furthermore, they could have been triggered by changes in the mental state during the prolonged exposure, occurring for example when the motion sickness rating had to be reported.

When asked about the actual motion afterwards, participants varied in their answers: some believed that the chair indeed had been rotating continuously during the test, while others believed that it had not. Perceived self-rotation, defined as the percentage of the time in full vection, was not positively correlated with the beliefs about self-rotation. This appears to contradict previous studies in which knowledge about the motion capabilities of the device was found to facilitate vection, either by shortening the vection onset time [8], or increasing the compellingness of the illusion [6, 7]. In these studies, however, the cognitive context was explicitly manipulated by comparing a “motion-possible” condition to a “motion-impossible” condition. This latter provides a level of certainty about the actual motion stimulus that was absent in our setup. Instead, our participants were deliberately shown that both drum and chair could be rotated independently from one another, thus creating an uncertainty about the motion stimulus. This could have predisposed them to the idea that their perception could differ from the actual motion. Such interpretation is also supported by the observation that, despite experiencing both periods of self-stationarity and drum-stationarity, the majority of the subjects answered affirmative to the question that either drum or chair had been rotating continuously during the experiment.

Interestingly, it was neither the intensity of the perceived motion itself, nor changes in the perceptual state, nor the belief or uncertainty about the actual motion stimulus which correlated highly with motion sickness intensity. Rather, it was the *difference* between perceived and believed motion. The combination of this difference and the individual motion sickness susceptibility explained 51% of the inter-subject variance in motion sickness intensity of the total group. The beliefs about the actual motion thus play an important part in this analysis. As mentioned above, the belief was not manipulated explicitly in different experimental conditions, but assessed using a post-stimulus questionnaire. This was done because we anticipated that the knowledge about the experimental device would lead to the stable belief that the perceived self-motion was indeed caused by rotation of the chair, and not of the drum in at least some participants. One could argue that this posterior assessment does not necessarily reflect the beliefs *during* the exposure. The fact that the question about the actual motion was asked, might have inclined subjects to answer differently from what they initially thought or felt. Although we cannot rule out this possibility playing a role in some participants, it is striking that the post-hoc question about certainty of actual drum rotation had–when compared to the perceived motion–such a predictive power in modeling motion sickness occurring prior to this question. This makes it unlikely that the posterior assessment of belief would be independent of the beliefs during the exposure.

Alternatively, one could also argue that the beliefs about the actual motion are shaped by the fact that sickness occurs, and not the other way around. In other words, that participants who became sick assumed that this had be due to the fact that they were fooled. This argumentation, however, assumes that participants have profound knowledge of the specific hypothesis of the study and link the motion sickness to a discrepancy between actual and experienced self-motion. We think this is very unlikely as no such information was provided; they could also have attributed motion sickness to the visual stimulation itself or link it to the intensity of self-rotation. Therefore, we think that the posterior assessment of the beliefs about actual self-motion can be used as a proxy for the beliefs during the stimulation. With that, our results highlight the importance of beliefs about one’s self-motion in the generation of motion sickness. Furthermore, it provides a possible explanation for the large inter-subject variability in motion sickness symptoms that is usually observed in optokinetic drum studies (e.g., [14–16]), as such a cognitive aspect was never part of the equation.

Our results fit well with the general framework of the sensory rearrangement theory [17], which acknowledges the discrepancy between the sensed and expected sensory signals as the cause of motion sickness. Instead of looking at the sensory signals itself–which are difficult to access–we looked at the self-motion percept that was derived from the integrated sensory signals and compared it to beliefs about the actual self-motion, the latter being a proxy for the expected self-motion. Although these beliefs might also have affected the self-motion percept itself, the discrepancy between perception and beliefs was still present and correlated with motion sickness induced by vection. Despite of the fact that our operationalization of the conflict is not exactly similar to the one used by Reason [17], this result sheds a different light on the nature of the expectancy component in the conflict. Reason argued that the expected sensory feedback stems from previous experience, i.e., through learning the sensory consequences of one’s actions. A vection stimulus consisting of an unfamiliar combination of sensory signals would result in sickness if it is not (yet) part of the recent exposure history [17]. Our findings suggest, however, that the nauseogenity was not related to the “inter-sensory conflict” in the motion stimulus, or the fact that the motion stimulus was unfamiliar. If that would have been the case, one would expect that especially changes in vection intensity would have been a significant predictor for motion sickness, as this entails a difference in perceived self-motion that is not corroborated by a matching vestibular signal. Similar to results of our previous study [18], however, the variability in vection intensity did not prove to be a significant predictor for motion sickness. Instead, motion sickness was related to the discrepancy between the believed and perceived motion. This suggests that the expected feedback is also under cognitive control, thus warranting a broader notion of expectancy than originally defined.

### Modeling cognitive influences in motion sickness models

A mathematical appraisal of the sensory re-arrangement theory has been provided by Oman [32], who applied concepts of observer theory to synthesize the derivation of the conflict responsible for motion sickness. The observer model stems from control theory and the central idea is that the state of the body cannot be derived directly from the–noisy–sensory signals; rather the state is predicted based on a copy of the command signals (“efference copy”), and a dynamical model of the controlled system (”internal model”). This predicted body state is thought to represent perceived self-motion. The expected sensory feedback is then derived from this predicted model state and a model of the sensors. The conflict between the actual and expected sensory feedback is used to steer the estimated body state towards reality, that is, the true body state, and it is this conflict that relates to motion sickness. As discussed by Mast & Ellis [33], it is likely that cognitive, top-down influences of sensory processing are related to the prediction mechanism of the model. The same authors recently proposed that the understanding of vestibular cognition could benefit from dynamic probabilistic models, which use Bayesian inference [34, 35]. Bayesian inference describes how the state is estimated based on noisy sensory data and prior expectations, and this technique has been applied in self-motion perception models [36–38]. The prior reflects the most common–and thus most likely–body state and is usually taken from previous experience. However, it can also stem from different sources, which makes it very suitable for modeling cognitive influences on vestibular processing. This was shown by Ellis and colleagues [35], who used dynamic Bayesian inference to illustrate how cognition could affect motion perception. They proposed that this cognitive influence could be utilized in rehabilitation for vestibular loss patients, to improve their motion perception using cognitive sources like mental imagery. As dynamic Bayesian models show similarities to certain classes of observer models [39], these principles could possibly be also applied to motion sickness models.

### Countermeasures

We demonstrated the role of beliefs in motion sickness using a visually induced motion illusion. As visually induced motion sickness is governed by the same underlying principles as other forms of motion sickness [40], we anticipate that our findings have implications for other forms of motion sickness as well, and opens up a new avenue for the development of countermeasures. The goal of such a cognitive intervention should be to bring the believed (i.e., expected) body state in line with the perceived body state, thereby minimizing a possible conflict. In essence, this is what anticipatory information does: it helps in improving the expected body state. This is illustrated by the observation that drivers usually do not suffer from sickness [41], and that a clear view of the road ahead helps the passenger in reducing sickness [42, 43]. Anticipatory information on the expected motion can also be given by instruction. For example, we hypothesize that telling participants that they would be physically rotated, without showing them the independent rotation of the drum on forehand, could strengthen their belief and perception about actual chair rotation, and thus reduce sickness. Another example where we expect that prior information could be helpful concerns motion sickness in high buildings [44]. Informing people that swaying motion of the building is to be expected may lower their prior belief that the building is–by definition–stationary, and thus affect the motion sickness conflict. In addition, we expect that these people might benefit from mental imagery. Mast and Ellis proposed mental imagery as a promising powerful tool in vestibular cognition [33–35]. Its potential effect on motion sickness is illustrated by the experiences of an astronaut, who tried to cope with motion sickness evoked by head tilts while rotating, also known as Coriolis cross coupling. Such head tilts evoke a strong illusion of tumbling and are very nauseogenic [45]. The astronaut explained that he was able to suppress the nausea when he imagined himself sitting on a fairground attraction that was performing the tumbling motion (Nooij, personal communication). In other words, he used mental imagery to alter his expected body state, and thereby reduced the discrepancy with what he perceived. Future research should be directed towards evaluating the usefulness of such techniques. If cognitive interventions are indeed able to shape the expected sensory feedback, they would offer a low-cost and efficient way to counteract motion sickness in situations where perception and reality diverge.

## Conclusion

The results of this study demonstrate that motion sickness induced by visual motion in otherwise stationary participants is correlated to the discrepancy between how the motion is perceived and what is believed to be actually occurring. These results hint at a crucial contribution of expectation in motion sickness and suggest that this contribution is under cognitive control. This opens up new possibilities to develop countermeasures.

## Supporting information

S1 Data Dataset containing all relevant individual data used for the analysis. See the manuscript text for explanation of variables.
(XLSX)
